# Dr. James Aloysius McMorrow

**Published:** 1917-05

**Authors:** 


**Dr. James Aloysius McMorrow,** Bellevue 1898, of Syracuse, died at the French Hospital in N. Y. city, March 12, following an operation for inflammation of the appendix, aged 40.

